# Studies on the Roles of Clathrin-Mediated Membrane Trafficking and Zinc Transporter Cis4 in the Transport of GPI-Anchored Proteins in Fission Yeast

**DOI:** 10.1371/journal.pone.0041946

**Published:** 2012-07-25

**Authors:** Wurentuya Jaiseng, Yue Fang, Yan Ma, Reiko Sugiura, Takayoshi Kuno

**Affiliations:** 1 Department of Pharmacology, School of Pharmaceutical Sciences, China Medical University, Shenyang, China; 2 Division of Molecular Pharmacology and Pharmacogenomics, Department of Biochemistry and Molecular Biology, Kobe University Graduate School of Medicine, Kobe, Japan; 3 Laboratory of Molecular Pharmacogenomics, School of Pharmaceutical Sciences, Kinki University, Higashi-Osaka, Japan; Simon Fraser University, Canada

## Abstract

We previously identified Cis4, a zinc transporter belonging to the cation diffusion facilitator protein family, and we demonstrated that Cis4 is implicated in Golgi membrane trafficking in fission yeast. Here, we identified three glycosylphosphatidylinositol (GPI)-anchored proteins, namely Ecm33, Aah3, and Gaz2, as multicopy suppressors of the MgCl_2_-sensitive phenotype of *cis4-1* mutant. The phenotypes of *ecm33*, *aah3* and *gaz2* deletion cells were distinct from each other, and Cis4 overexpression suppressed Δ*ecm33* phenotypes but did not suppress Δ*aah3* defects. Notably, green fluorescent protein-tagged Ecm33, which was observed at the cell surface in wild-type cells, mostly localized as intracellular dots that are presumed to be the Golgi and endosomes in membrane-trafficking mutants, including Δ*apm1*, *ypt3-i5*, and *chc1-1* mutants. Interestingly, all these membrane-trafficking mutants showed hypersensitivity to BE49385A, an inhibitor of Its8 that is involved in GPI-anchored protein synthesis. Taken together, these results suggest that GPI-anchored proteins are transported through a clathrin-mediated post-Golgi membrane trafficking pathway and that zinc transporter Cis4 may play roles in membrane trafficking of GPI-anchored proteins in fission yeast.

## Introduction

Glycosylphosphatidylinositol (GPI) anchoring is a common post-translational lipid modification by which proteins are attached to the cell surface in all eukaryotic cells. GPI-anchored proteins are functionally diverse and are important for signal transduction, cell-cell interaction, cell adhesion, cell surface protection, and cell wall synthesis [1], [2], [3], [4]. In mammalian cells, more than 150 proteins including receptors, adhesion molecules, and enzymes, are reportedly linked by GPI anchor [5], [6]. In budding yeast *Saccharomyces cerevisiae*, more than 60 genes are predicted to encode GPI-anchored proteins that play important roles in cell wall biogenesis and cell wall assembly [7], [8]. In the fission yeast *Schizosaccharomyces pombe*, 33 GPI-anchored protein candidates have been identified among 4950 *S. pombe* ORFs [9].

We have been studying the role of calcineurin in fission yeast *S. pombe*, because this system is amenable to genetic analysis and has many advantages in terms of its relevance to higher systems. In our previous study, we identified a mutation in the *its8* ^+^ gene encoding a homolog of the budding yeast Mcd4p and human Pig-n that are involved in GPI anchor synthesis through a genetic screen using the immunosuppressant drug FK506, a specific inhibitor of calcineurin [10].

In another screen using FK506, we identified a mutant allele of the *cis4* ^+^ gene that encodes a zinc transporter belonging to the cation diffusion facilitator (CDF) protein family, and we characterized the role of Cis4 in Golgi membrane trafficking in fission yeast [11]. In order to gain further insight into the function of Cis4, we screened for multicopy suppressors of the MgCl_2_-sensitive phenotype of the *cis4-1* mutant cells and identified three genes encoding GPI-anchored proteins, namely Ecm33, Aah3, and an uncharacterized protein, Gaz2.

The *ecm33* ^+^ gene was previously identified as a target of the two transcription factors Atf1 and Mbx1 and is involved in the negative feedback regulation of Pmk1 cell integrity signaling [12]. The *aah3* ^+^ gene encodes an α-amylase homolog required for cell wall integrity, morphogenesis and vacuolar protein sorting [13], [14]. These three GPI-anchored proteins all suppressed the phenotypes of *cis4-1* mutant cells. Furthermore, we showed that GFP-Ecm33 localized at the cell surface in wild-type cells, whereas it mostly localized as intracellular dots which are presumed to be the Golgi and endosomes in membrane-trafficking mutants, including Δ*apm1*, *ypt3-i5*, and *chc1-1* mutants. Taken together, these results highlight the importance of the clathrin-mediated post-Golgi membrane trafficking pathway as well as the zinc transporter Cis4 in the intracellular transport of GPI-anchored proteins.

## Results

### Isolation of the *ecm33* ^+^, *aah3* ^+^, and *gaz2* ^+^ genes as multicopy suppressors of zinc transporter *cis4-1* mutant

**Figure 1:**
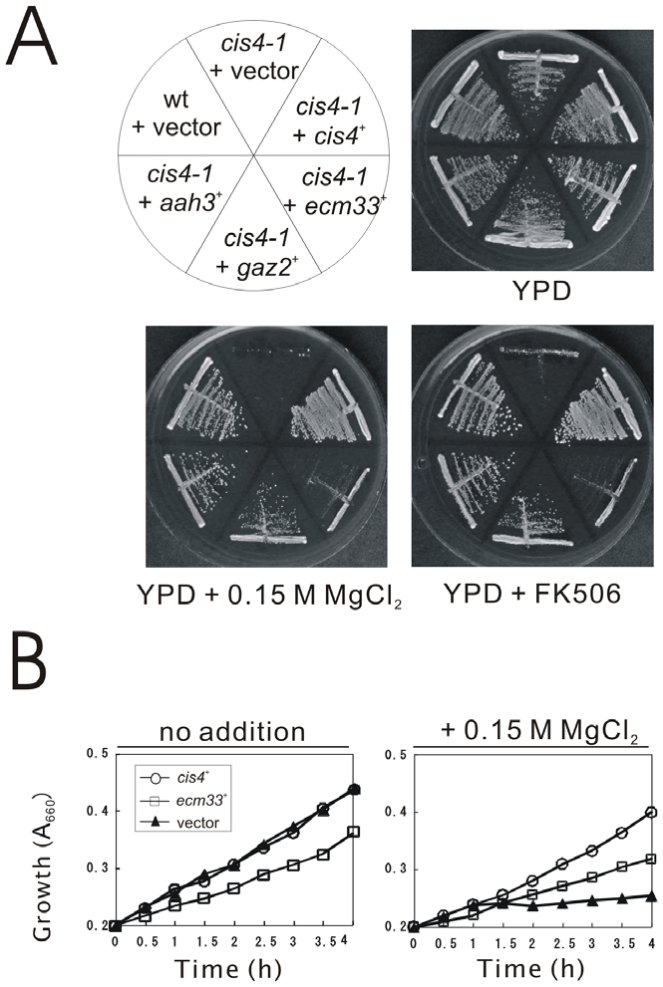
Isolation of Ecm33, Aah3, and Gaz2 as multicopy suppressors of the *cis4-1* mutant cells. (A) The *cis4-1* mutant cells were transformed with either the pDB248 multicopy vector or the vector containing *ecm33* ^+^, *aah3* ^+^, or *gaz2* ^+^. Cells were then streaked onto plates containing YPD, YPD plus 0.15 M MgCl_2_, or YPD plus 0.5 µg/ml FK506 and then incubated for 4 days at 30°C. (B) The *cis4-1* mutant cells were transformed with either the pDB248 multicopy vector (closed triangles), or the vector containing *cis4* ^+^ (open circles) or *ecm33* ^+^ (open squares). Cells were then diluted with fresh EMM or EMM plus 0.15 M MgCl_2_ and incubated at 30°C. Growth was recorded by measurement of the absorbance at 660 nm. Data were averaged from three independent experiments, each sample done in duplicate.

We have previously demonstrated that Cis4 is a zinc transporter belonging to the CDF protein family, and plays a role in Golgi membrane trafficking in fission yeast [11]. To better understand the function of Cis4, we screened for genes that when overexpressed could suppress the Cl^−^ hypersensitivity of *cis4-1* mutant. The *cis4-1* mutant cells grew well in rich YPD medium, however, in the presence of 0.15 M MgCl_2_, the *cis4-1* cells failed to grow, whereas wild-type cells grew well (Figure 1A). Notably, overexpression of the *ecm33* ^+^ gene partially suppressed the MgCl_2_ sensitivity of *cis4-1* mutant, and overexpression of the *aah3* ^+^ and *gaz2* ^+^ genes more strongly suppressed the MgCl_2_ sensitivity of the *cis4-1* mutant (Figure 1A). Then we further determined the growth rates of *ecm33* ^+^ overexpression in liquid media to assess the level of the suppression of the phenotype. Consistently, results showed that *cis4-1* mutant cells harboring the multicopy vector grew almost normally but stopped growing 1 h after shift to the YPD media containing 0.15 M MgCl_2_. However, the *cis4-1* mutant cells expressing *ecm33* ^+^ gene could grew in the presence of 0.15 M MgCl_2_, although the growth was slower than that of the *cis4-1* mutant cells harboring *cis4* ^+^ gene (Figure 1B). Likewise, these three genes complemented the FK506-sensitive phenotype of the *cis4-1* mutant (Figure 1A). Then we examined in Δ*cis4* deletion mutants the effects of the overexpression of *ecm33* ^+^, *aah3* ^+^, and *gaz2* ^+^ genes, respectively, and results showed that these genes also suppressed the MgCl_2_-sensitive and FK506-sensitive growth defect of the Δ*cis4* cells (our unpublished data).

The *ecm33* ^+^ gene encodes a 43.3 kDa protein (Ecm33) comprising 421 amino acids and containing a signal peptide for GPI anchor in its N-terminus. The *aah3* ^+^ gene encodes a cell surface GPI-anchored protein (Aah3) consisting of 564 amino acids (63.2 kDa) and containing an alpha-amylase domain as well as a DUF1966 domain of unknown function. The *gaz2* ^+^ gene, based on the nucleotide sequence determination, encodes a conserved fungal protein of 317 amino acids. Notably, the N-terminal portion of Gaz2 contains an amino acid signal sequence, and in addition, the *gaz2* ^+^ gene product (Gaz2) has a serine-rich region. These three proteins are conserved in fungi, and Ecm33 is structurally similar to the budding yeast Pst1p and Ecm33p, while Aah3 and Gaz2 have no apparent *S. cerevisiae* ortholog. The amino acid sequence similarity among Ecm33, Gaz2, and Aah3 are considerably low, and the domain structure is distinct from each other. Ecm33 is a member of the Ecm33/Sps2 family, Aah3 is an alpha-amylase protein, and Gaz2 seems to be a non-enzymatic serine-rich cell wall protein. The only structural thing they have in common is that they contain signal peptides for ER entry and GPI anchoring. Probably, a common feature of these three proteins is that they are highly glycosylated, and the suppression is related to their glycosylation onto the proteins. As the feature of these three proteins is their high glycosylation, the suppression might be due to an indirect effect of overexpressing the GPI proteins.

### Phenotypes of *ecm33* ^+^, *gaz2* ^+^, and *aah3* ^+^ deletion mutants

**Figure 2:**
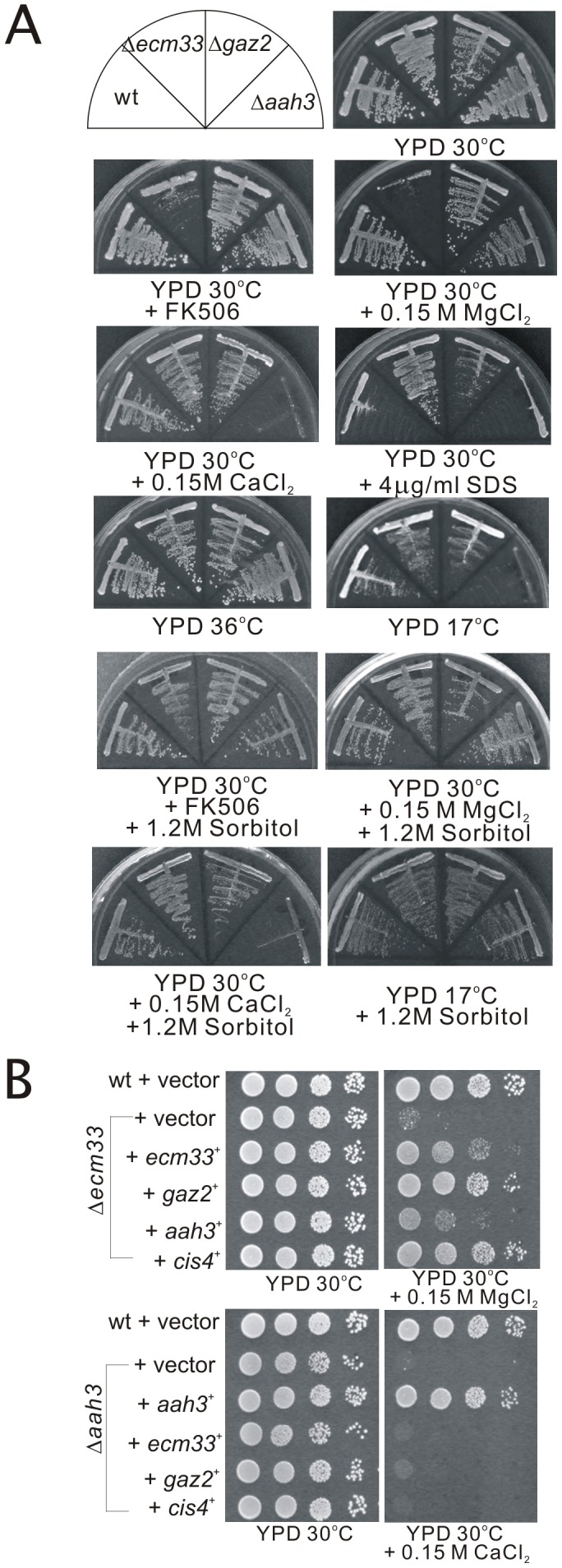
The Δ*ecm33*, Δ*aah3*, and Δ*gaz2* mutants displayed distinct phenotypes. (A) Phenotypes of the Δ*ecm33*, Δ*aah3*, and Δ*gaz2* mutants. Upper panel, Cells were streaked onto each plate as indicated, and then incubated at 30°C for 4 days, at 36°C for 3 days or at 17°C for 7 days. Lower panel, MgCl_2_-sensitive and FK506-sensitive phenotypes of Δ*ecm33* and cold temperature-sensitive phenotype of Δ*aah3* were osmoremedial, whereas CaCl_2_-sensitive phenotype of Δ*aah3* was not. Cells were streaked onto each plate as indicated, and then incubated at 30°C for 4 days or at 17°C for 7 days. (B) Effects of overexpression of the *gaz2* ^+^, *aah3* ^+^, and *cis4* ^+^ genes on the phenotypes of Δ*ecm33*, and effects of overexpression of the *ecm33* ^+^, *gaz2* ^+^, and *cis4* ^+^ genes on the phenotypes of Δ*aah3*. Wild-type cells, Δ*ecm33* or Δ*aah3* cells transformed with a control vector or the vector containing *ecm33* ^+^, *gaz2* ^+^, *aah3* ^+^, and *cis4* ^+^ were spotted onto YPD plates or YPD plus 0.15 M MgCl_2_ and incubated at 30°C for 4 days.

We constructed a null mutation in the *ecm33* ^+^ and *gaz2* ^+^ genes, respectively (see Materials and Methods) and found that the *gaz2* deletion mutant was also viable (Figure 2A, upper panel), indicating that Gaz2 is not essential for cell viability. Then we compared the phenotypes of *ecm33* ^+^, *gaz2^+^*, and *aah3^+^* gene deletion mutants. With regard to the *cis* phenotypes including FK506 sensitivity and MgCl_2_ sensitivity [11], Δ*ecm33* cells exhibited sensitivity to both FK506 and MgCl_2_, whereas the Δ*gaz2* and Δ*aah3* cells were not sensitive to FK506 or MgCl_2_. With regard to CaCl_2_ sensitivity, Δ*aah3* cells failed to grow on YPD plate containing 0.15 M CaCl_2_, whereas Δ*ecm33* and Δ*gaz2* cells grew well on the same plate. With regard to temperature sensitivity, Δ*aah3* cells were very sensitive to cold temperature while the others were not sensitive, and all were not sensitive to high temperature. With regard to the altered sensitivity to the plasma membrane perturbing agent, sodium dodecyl sulfate (SDS), Δ*ecm33* and Δ*gaz2* but not Δ*aah3* cells were significantly more resistant to SDS as compared with that of the wild-type cells (Figure 2A, upper panel).

Because some of the GPI-anchored proteins were found to be involved in cell wall integrity [15], we then examined whether the phenotypes of these three GPI-anchored protein mutants were suppressible by osmotic stabilization of the medium with sorbitol. Our results showed that in Δ*ecm33* cells, sorbitol suppressed the FK506 sensitivity and MgCl_2_ sensitivity of the cells. In Δ*aah3* cells, sorbitol suppressed the cold temperature sensitivity of the cells, whereas sorbitol failed to suppress the CaCl_2_ sensitivity of the cells (Figure 2A, lower panel). Consistent with these results, Morita *et al* showed that the morphological defect of Δ*aah3* cells were not rescued in the presence of 1.2 M sorbitol-YES medium [13].

### Analysis of the overlapping functions among the three GPI-anchored proteins

As described above, the domain structures of Ecm33, Aah3, and Gaz2 are distinct, therefore it seems likely that *ecm33* ^+^, *aah3* ^+^ and *gaz2* ^+^ are not functionally redundant. To test this possibility, we examined the effects of the overexpression of *gaz2* ^+^ and *aah3* ^+^ on the phenotypes of the *ecm33* ^+^ deletion mutants, as well as the overexpression of *ecm33* ^+^ and *gaz2* ^+^ on the phenotypes of the *aah3* ^+^ deletion mutants. As shown in Figure 2B, the results showed that overexpression of *gaz2* ^+^, but not *aah3* ^+^, suppressed the MgCl_2_-sensitive growth defect of the *ecm33* ^+^ gene deletion mutants. On the other hand, overexpression of the *ecm33* ^+^ or *gaz2* ^+^ genes failed to suppress the phenotypes of the *aah3* ^+^ deletion mutants. Thus, these findings suggest that the structures of the three GPI-anchored proteins are distinct from each other, and that these three proteins have only partial overlapping functions. We also examined the effects of the overexpression of *cis4* ^+^ on the phenotypes of the Δ*ecm33* and Δ*aah3* mutants. The results showed that overexpression of *cis4* ^+^ suppressed the MgCl_2_-sensitive growth defect of the Δ*ecm33* mutants, but failed to suppress the CaCl_2_-sensitive phenotype of the Δ*aah3* mutants (Figure 2B).

### Deletion analysis of the *ecm33* ^+^ gene

**Figure 3:**
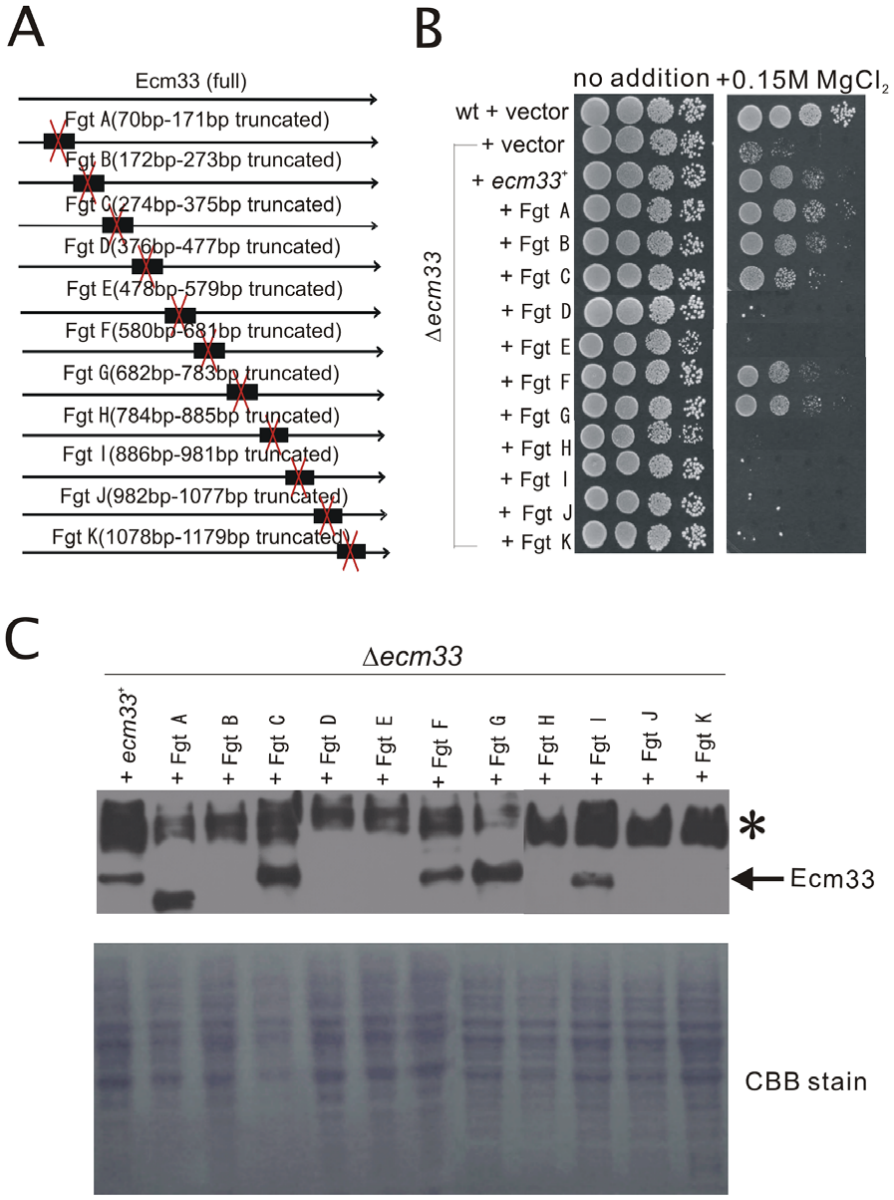
Deletion analysis of the *ecm33* ^+^. (A) Structural features of the truncated mutants of Ecm33. (B) The Δecm33 mutant phenotype suppression by Ecm33 truncated mutants. Cells transformed with the multicopy vector or vector containing various truncated genes were spotted onto each plate as indicated and incubated for 4 days at 30°C. (C) Protein levels of Ecm33 examined by immunoblot analysis. The ecm33 deletion cells were transformed with the *ecm33* ^+^, or truncated *ecm33* ^+^ gene were grown to mid-log phase in EMM at 30°C. Cells were washed and incubated for 24 h and then analyzed by immunoblotting using anti-Ecm33 monoclonal antibody as described under “Materials and Methods.” The panel indicated as CBB stain shows the CBB staining of the same gel to show the presence of equal amount of proteins in each lane.

To determine the functional region of Ecm33, we prepared a series of truncated forms of Ecm33. Structural feature of the deletion mutants employed in this study is illustrated in Figure 3A. Results showed that in Δ*ecm33* mutants, the overexpression of the full-length Ecm33 as well as Ecm33 fragment A, fragment B, fragment C, fragment F, and fragment G suppressed the phenotypes of the mutants (Figure 3B). However, overexpression of Ecm33 fragment D, fragment E, fragment H, fragment I, fragment J, and fragment K failed to suppress the phenotypes of Δ*ecm33* mutants (Figure 3B). Overexpression of these truncated versions of Ecm33 showed similar genetic suppression profile of the *cis4-1* mutant as compared with that of the Δ*ecm33* cells (our unpublished data).

We also examined the protein levels of truncated mutants of Ecm33 in the Δ*ecm33* cells by immunoblotting with anti-Ecm33 monoclonal antibody [12] (Figure 3C). The immunoblot analysis detected an appreciable amount of Ecm33 fragments A, C, F, G, and I, but failed to detect fragment B, D, E, H, J or K. These results are consistent with the above results that overexpression of Ecm33 fragments A, C, F, G except for fragment I suppressed the phenotypes of the Δ*ecm33* cells. In addition, the Ecm33 fragment B was not detected in the cells by immunoblotting, although overexpression of the fragment B suppressed the phenotypes of the Δ*ecm33* cells. It is possible that the Ecm33 fragment B contains the epitope for the monoclonal antibody. The reasons for the inability of the antibody to detect other Ecm33 fragments as well as the functional importance of these fragments are unknown.

### Subcellular localization of Ecm33 and Gaz2

**Figure 4:**
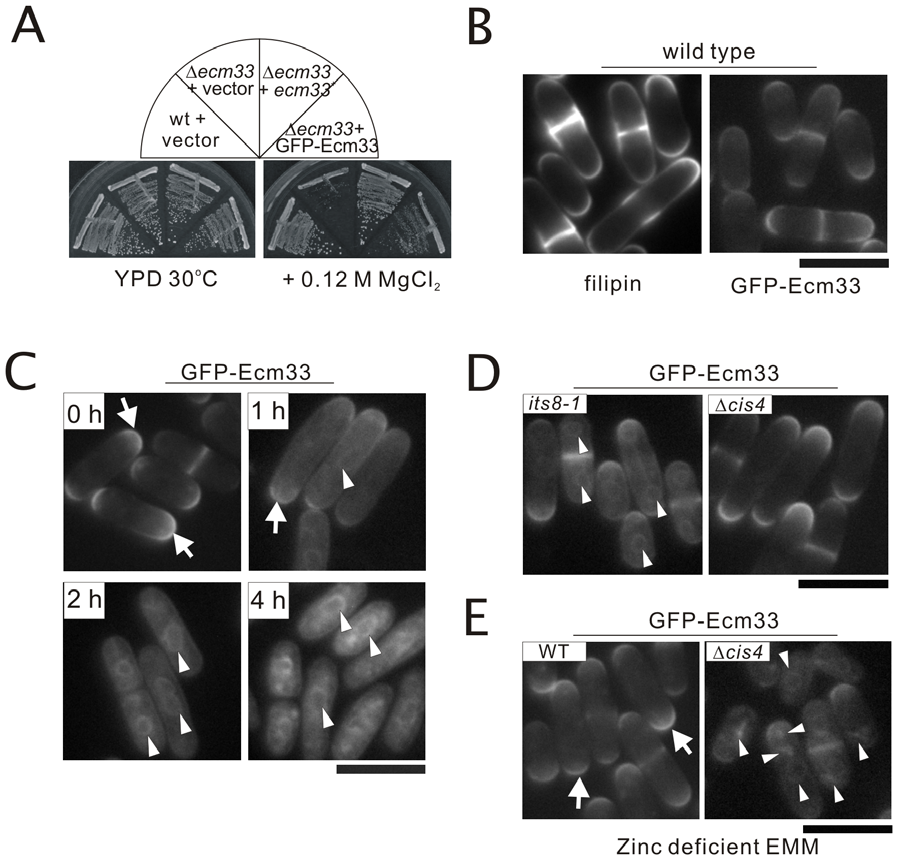
Subcellular localization of GFP-Ecm33. (A) GFP-tagged Ecm33 was functionally similar to the non-tagged protein. Cells transformed with the multicopy vector harboring gene encoding GFP-Ecm33 or the empty vector was streaked onto each plate containing YPD or YPD plus 0.12 M MgCl_2_, and then incubated for 4 days at 30°C. (B) GFP-Ecm33 localized to the cell surface or medial regions in wild-type cells. Wild-type cells expressing chromosome-borne GFP-Ecm33 were cultured in EMM medium at 27°C, and were examined by fluorescence microscopy; Sterol localization detected by filipin staining (see Materials and Methods) in wild-type cells. Bar: 10 µm. (C) GFP-Ecm33 localized to the structure surrounding the nucleus in wild-type cells when cells were treated with BE49385A. Wild-type cells expressing chromosome-borne GFP-Ecm33 were cultured in EMM medium and incubated at 27°C, and were examined by fluorescence microscopy at 0 hour, 1 hour, 2 hour, and 4 hour after 1 µg/ml BE49385A was added to the medium. Bar: 10 µm. (D) GFP-Ecm33 primarily localized to the structure surrounding the nucleus and to the cell surface in *its8-1* mutant, while the localization of GFP-Ecm33 was normal in Δ*cis4* mutant. The *its8-1* mutant and Δ*cis4* mutant expressing chromosome-borne GFP-Ecm33 were cultured in EMM medium and incubated at 27°C, and were examined by fluorescence microscopy. Bar: 10 µm. (E) In the zinc-deficient medium GFP-Ecm33 primarily localized to the intracellular dots and to the ER in Δ*cis4* cells. The wild-type cells and Δ*cis4* mutant expressing chromosome-borne GFP-Ecm33 were cultured in the zinc deficient EMM medium at 27°C for 48 hours, and were examined by fluorescence microscopy. Bar: 10 µm.

In order to investigate the subcellular localization and membrane trafficking of the Ecm33 and Gaz2 protein, plasmids carrying GFP-Ecm33 and GFP-Gaz2 fusions, respectively, were constructed. On Ecm33, a GFP carrying the S65T mutation was inserted into 60 bp from the N-terminus of Ecm33. Fujita *et al* demonstrated that HA- and mRFP-tagged versions of Gas1, a well-characterized GPI-anchored protein in *Saccharomyces cerevisiae*, were generated by inserting the tags in Gas1 immediately following the N-terminal signal sequence and both the tagged proteins were functional [16]. Therefore, we constructed GFP-Ecm33 fusion by inserting the GFP tag immediately following the N-terminal signal sequence. Results showed that the construct of GFP-Ecm33 was functional as cells expressing GFP-Ecm33 suppressed the phenotypes of Δ*ecm33* mutants (Figure 4A). Then, we examined the localization of GFP-Ecm33 expressed from its own promoter in wild-type cells, and results showed that GFP-Ecm33 localized to the cell surface or the medial regions. This observation was similar to the sterol localization of filipin fluorescence that was enriched in the plasma membrane at the growing cell tips and at the site of cytokinesis (Figure 4B). Also, this finding was consistent with the data obtained using anti-Ecm33 antibody by immunofluorescence microscopy [12]. On Gaz2, a GFP was inserted into 600 bp from the N-terminus of Gaz2 because deletion of 500–600 bp of Gaz2 gene did not affect its suppression ability on the phenotype of *cis4-1* mutant. However, the construct was not functional and cells expressing GFP-Gaz2 failed to suppress the phenotypes of the *cis4-1* mutant (our unpublished data). Presumably, this is because the GFP tag is inserted somewhere in the middle of the Gaz2 protein.

Next, we examined the effect of BE49385A, an inhibitor of Its8, on the subcellular localization of GFP-Ecm33 in wild-type cells. We previously identified a mutation in the *its8* ^+^ gene that are involved in GPI anchor synthesis, and showed that Its8 is a molecular target of BE49385A [10]. In wild-type cells, before the addition of BE49385A as shown in Figure 4C, GFP-Ecm33 localized to the cell surface or the medial regions. Then, 1 hour after the addition of 1 µg/ml BE49385A to the medium, GFP-Ecm33 mostly localized at the cell surface (Figure 4C, arrowheads) and also localized at the structure surrounding the nuclei that are considered to be the endoplasmic reticulum (ER) (Figure 4C, arrows). Then, 2 hours or 4 hours after treatment with BE49385A, GFP-Ecm33 mostly localized to the nuclear envelope and the peripheral ER rather than the cell surface (Figure 4C, arrows). In *its8-1* mutant cells, the subcellular localization of GFP-Ecm33 was also examined. As expected, GFP-Ecm33 primarily localized to the ER and to the cell surface in the *its8-1* mutant cells (Figure 4D, arrows), suggesting that the impairment of GPI anchor synthesis caused the defective attachment of GPI-anchor to the Ecm33 protein thereby resulting in the abnormal GFP-Ecm33 localization in the ER. Then we also examined the subcellular localization of GFP-Ecm33 in the Δ*cis4* cells, and results showed that GFP-Ecm33 was observed at the cell surface or the medial region in the Δ*cis4* cells (Figure 4D) similar to that observed in the wild-type cells (Figure 4B). We further examined the effect of zinc deficiency on the subcellular localization of GFP-Ecm33 in the Δ*cis4* cells by removing zinc from the EMM medium. As shown in Figure 4E, the fluorescence of GFP-Ecm33 in wild-type cells was enriched in the cell surface and the medial region in the zinc-deficient medium. In contrast, in Δ*cis4* cells GFP-Ecm33 primarily localized to the intracellular dots and to the ER in the zinc-deficient medium (Figure 4E).

### Genetic interaction between *cis4* ^+^ and *its8* ^+^ genes

**Figure 5:**
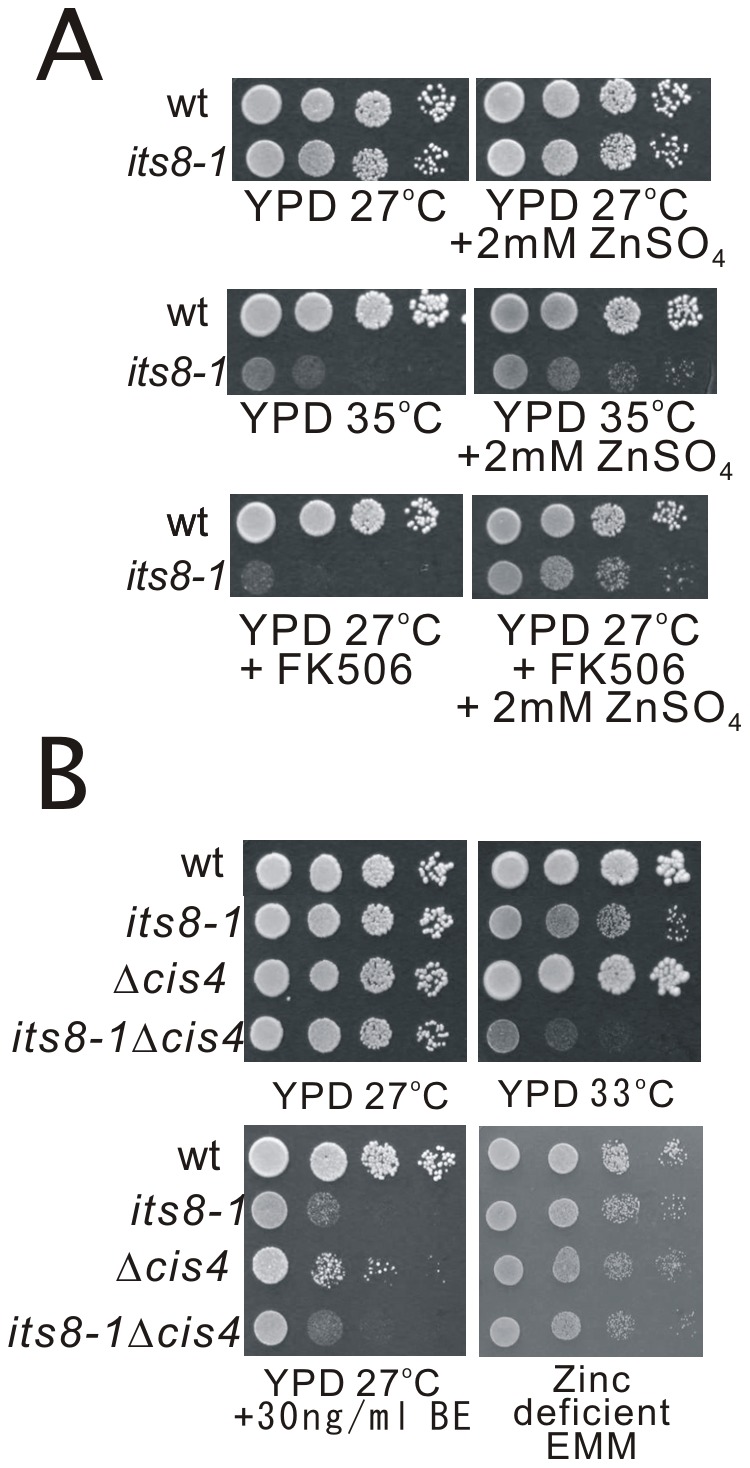
Genetic interaction between *cis4^+^* and *its8^+^* genes. (A) Effect of the addition of extracellular Zn^2+^ on the phenotypes of *its8-1* mutant. Wild-type or *its8-1* mutant cells were spotted onto each plate as indicated and then incubated for 4 days at 27°C or at 35°C. (B) The *its8-1*Δ*cis4* double mutants showed more marked temperature sensitivity than the single mutants, while the double mutants showed similar BE49385A-sensitivity as compared with that of the *its8-1* mutant. Wild-type, *its8-1*, Δ*cis4*, and *its8-1*Δ*cis4* cells were spotted onto each plate as indicated and then incubated for 4 days at 27°C or at 33°C.

Then we examined the effect of Zn^2+^ on the phenotypes of the *its8-1* mutant cells. The results showed that the addition of Zn^2+^ to the medium significantly rescued the high temperature-sensitive and FK506-sensitive phenotypes of the *its8-1* mutant (Figure 5A).

Our previous study suggested that Cis4 localizes to the *cis*-Golgi and was involved in Golgi membrane trafficking through regulating the zinc homeostasis [11]. In order to investigate the functional relationship between Cis4 and Its8, we constructed *its8-1*Δ*cis4* double mutants, and examined the effect of temperature, BE49385A, and zinc deficiency respectively on these cells. On the effect of temperature, in the *its8-1*Δ*cis4* double mutants, these cells exhibited more marked temperature sensitivity than that of the *its8-1* single mutants (Figure 5B), suggesting that there is a genetic interaction between Its8 and Cis4. On the effect of BE49385A, in the Δ*cis4* cells interestingly, the growth of these single deletion cells was significantly inhibited by BE49385A as compared with that of the wild-type cells, although the sensitivity of the Δ*cis4* cells was not as severe as that of the *its8-1* mutant (Figure 5B). In the *its8-1*Δ*cis4* double mutants, notably, these cells exhibited the BE49385A-sensitive growth defects similar to that of the *its8-1* single mutant. On the effect of zinc deficiency, in the *its8-1*Δ*cis4* double mutants interestingly, these cells were observed to have a very small colony size similar to that of the Δ*cis4* single mutant in the zinc-deficient medium (Figure 5B). These results suggest that the impairment of GPI-anchor synthesis and zinc-ion homeostasis in the double mutant is similar to that of *its8-1* and Δ*cis4* single mutants, respectively.

Furthermore, we examined the effect of overexpressed multicopy suppressors of the *cis4-1* mutant on the MgCl_2_-sensitive phenotypes of the *apm1-1* mutant, an allele of *apm1* ^+^ gene that encodes μ1A subunit of the clathrin-associated adaptor protein complex 1(AP-1) implicated in the Golgi/endosome function [17]. The multicopy suppressors of the *cis4-1* mutant identified here were the three genes encoding GPI-anchored protein namely *ecm33* ^+^, *aah3* ^+^ and *gaz2* ^+^, and in addition, two other multicopy suppressor genes including *pmp1* ^+^, and SPCC1322.03 (*trp1322* ^+^) that also suppressed the MgCl_2_-sensitive phenotype of *cis4-1* mutant (Materials and Methods). The *pmp1* ^+^ gene encodes a dual-specificity MAPK phosphatase that negatively regulates the Pmk1 MAPK signaling [18]. The *trp1322* ^+^ gene encodes transient receptor potential (TRP)-like ion channel that mediates the cytoplasmic Ca^2+^ rise caused by the extracellularly added CaCl_2_ [19]. As shown in Table 1, all the multicopy suppressors with the exception of the *aah3* ^+^ gene significantly suppressed the MgCl_2_-sensitive phenotype of the *apm1-1* mutant.

**Table 1:** Complementation of the MgCl_2_-sensitive phenotype of the *cis4-1* mutant and *apm1-1* mutant.

Plasmid	Complementation of the mutants	
	*cis4-1*	*apm1-1*
*ecm33* ^+^	++	++
*aah3* ^+^	++	−
*gaz2* ^+^	++	++
*pmp1* ^+^	++	+
*trp1322^+^*	++	++

Note: Each transformant, carrying various genes on the multicopy plasmids, was streaked onto YPD plates in the presence or absence of MgCl_2_ and incubated at 27°C for 4 days. ++, complemented the 0.15 M MgCl_2_-sensitive phenotype; +, complemented the 0.12 M MgCl_2_-sensitive phenotype; −, did not complement.

### Localization of GFP-Ecm33 in various membrane trafficking mutants

**Figure 6:**
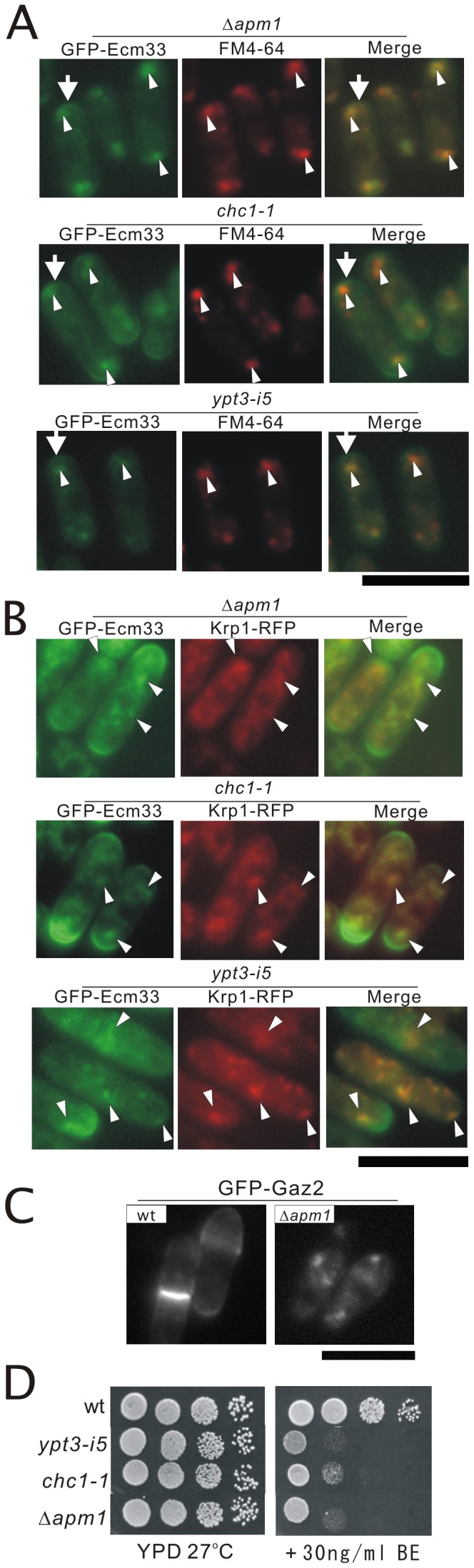
Subcellular localization of GFP-Ecm33 in membrane trafficking mutants. (A) The Apm1-deletion (Δ*apm1*), *chc1-1* mutant and *ypt3-i5* mutant expressing chromosome-borne GFP-Ecm33 cultured in EMM medium were incubated with FM4-64 fluorescent dye for 5 min at 27°C to visualize Golgi/endosomes. GFP-Ecm33 localization and FM4-64 fluorescence were examined under a fluorescence microscope. Bar: 10 µm. (B) Localization of GFP-Ecm33 and Krp1-RFP in the membrane trafficking mutants. The Apm1 deletion (Δ*apm1*), *chc1-1* mutant and *ypt3-i5* mutant expressing chromosome-borne GFP-Ecm33 were transformed with the pREP1 vector expressing Krp1 fused to RFP at its C-terminus. Cells were grown to early log phase in EMM containing 4 µM thiamine at 27°C, and were examined by fluorescence microscopy. Bar: 10 µm. (C) GFP-Gaz2 localized to the cell surface or medial regions in wild-type cells, while GFP-Gaz2 localized to the intracellular dots in the Δ*apm1* cells. Cells transformed with the multicopy vector GFP-Gaz2 were cultured in EMM medium at 27°C, and were examined by fluorescence microscopy. Bar: 10 µm. (D) All the membrane trafficking mutants tested namely *ypt3-i5*, *chc1-1*, and Δ*apm1* mutants showed hypersensitivity to BE49385A. Wild-type, *ypt3-i5*, *chc1-1*, and Δ*apm1* cells were spotted onto each plate containing YPD or YPD plus 30 ng/ml BE49385A as indicated, and then incubated at 27°C for 4 days.

As shown above, the overexpression of the GPI-anchored proteins suppressed the MgCl_2_ sensitivity of the mutant allele of the *apm1* ^+^ gene. This prompted us to hypothesize that Apm1 may play roles in membrane trafficking of the GPI-anchored proteins. Then, we examined the localization of GFP-Ecm33 in Δ*apm1* cells. In wild-type cells, GFP-Ecm33 clearly localized at the cell surface and the medial regions as shown in Figure 4B. In Δ*apm1* cells, in contrast, GFP-Ecm33 primarily localized as dot-like structures that were observed in the cytoplasm (Figure 6A, arrows) as well as at the cell surface and the division site (Figure 6A, arrowheads). Next, we examined the localization of GFP-Ecm33 in other membrane trafficking mutants namely, *ypt3-i5* mutant and *chc1-1* mutant. The Rab/Ypt GTPase Ypt3 has been implicated in the membrane trafficking associated with the Golgi complex, and its mutation confers sensitivity to FK506 and defects in cell wall integrity [20]. The *chc1* ^+^ gene encodes clathrin heavy chain Chc1 involved in intracellular protein transport. Results showed that the *chc1-1* mutant exhibited *its* (*its* for *i*mmunosuppressant- and *t*emperature-*s*ensitive) phenotype [21] (Figure S1A). Sequence analysis of the genomic DNA from the *chc1-1* mutant revealed that arginine at 1615 was mutated to a termination codon by a C-to-T transition (CGA to TGA), and resulted in a truncated protein product lacking 51 amino acids downstream of the mutation (Figure S1B). In the membrane trafficking mutants including *chc1-1* mutant and *ypt3-i5* mutant, results showed that GFP-Ecm33 also localizes as intracellular dot-like structures (Figure 6A, arrows) in addition to the cell surface and the division site (Figure 6A, arrowheads). So, we examined whether the dot-like fluorescence of GFP-Ecm33 co-localized with the endocytic tracer dye FM4-64 during an early stage of endocytosis in Δ*apm1*, *chc1-1* mutant and *ypt3-i5* mutants. After 5 min of dye uptake, most of the GFP-Ecm33 dot-like structures co-localized with FM4-64-positive structures in Δ*apm1*, *chc1-1* mutant and *ypt3-i5* mutants (Figure 6A, Merge). This strongly suggests that the intracellular dot-like fluorescence of GFP-Ecm33 represents Golgi/endosome compartments. Then we further examined the co-localization of GFP-Ecm33 with Krp1 fused to monomeric red fluorescent protein (RFP) at its C-terminus. Krp1 is a furin/Kex2 homolog that resides in the Golgi/endosome [22], [23]. As shown in Figure 6B, intracellular GFP-Ecm33 mostly co-localized with Krp1-RFP (Figure 6B). Thus, GFP-Ecm33 localized at Golgi/endosome structures in addition to the cell surface and the division site in these mutants, suggesting that GPI-anchored proteins were not correctly transported and were retained at the Golgi/endosome structures in these membrane trafficking mutants. Similarly, in the wild-type cells, GFP-Gaz2 also clearly localized at the cell surface and medial regions (Figure 6C), while in Δ*apm1* cells, GFP-Gaz2 localized as intracellular dot-like structures (Figure 6C). Interestingly, all of the membrane trafficking mutants that were tested showed hypersensitivity to BE49385A (Figure 6D).

## Discussion

Here, we identified three genes encoding GPI-anchored proteins, namely Ecm33, Aah3, and Gaz2 as multicopy suppressors of the MgCl_2_-sensitive and FK506-sensitive phenotypes of the *cis4-1* mutant. Furthermore, we suggest that GPI-anchored proteins are transported through a clathrin-mediated post-Golgi membrane trafficking pathway in fission yeast. To our knowledge, this is the first report that characterized the roles of clathrin-mediated post-Golgi membrane trafficking pathway as well as the zinc transporter Cis4 in membrane trafficking of GPI-anchored proteins in fission yeast.

### GPI-anchored proteins and clathrin-mediated post-Golgi membrane trafficking

Important finding of this study is the role of clathrin-mediated post-Golgi membrane trafficking pathway in the transport of GPI-anchored proteins in fission yeast. In budding yeast, GPI-anchored proteins are transported from the ER to the Golgi apparatus in vesicles distinct from those containing non-GPI-anchored proteins such as the general amino acid permease Gap1p and pro-alpha factors [24], [25], [26], and the transport of GPI-anchored proteins and non-GPI-anchored proteins from the trans-Golgi network (TGN) to the plasma membrane is also regulated by different sorting and packaging machinery [27]. Consistent with these, Castillon *et al* observed that GPI-anchored proteins accumulate in ER exit sites (ERES) that are distinct from those in which other secretory proteins accumulate [28]. More recently, Rivier *et al* reported that in mammalian cells, GPI-anchored and other secretory proteins are not segregated upon exit from the ER, in contrast to the remarkable segregation seen in budding yeast [29].

In this study in fission yeast, we observed the subcellular localization of a GPI-anchored protein Ecm33 using GFP fusion proteins *in vivo*. Results showed the abnormal localization of GFP-Ecm33 in all of the membrane trafficking mutants tested including *ypt3-i5*, *chc1-1*, and Δ*apm1* mutants. The AP-1 complex that links a clathrin to the membrane plays a role in the post-Golgi membrane trafficking including exit transport from the TGN to endosomes, endosomes to the TGN, and TGN or endosomes to the plasma membrane [23]. In the Δ*apm1* and *chc1-1* mutants, GFP-Ecm33 was primarily seen as dot-like structures presumed to be the Golgi/endosomes, suggesting the delay in the clathrin-mediated post-Golgi membrane trafficking of GPI-anchored protein Ecm33 to the cell surface in these membrane trafficking mutants. Moreover, GFP-Ecm33 also localized at Golgi/endosome structures in addition to the cell surface and the division site in the *ypt3-i5* mutant. Ypt3 is involved at multiple steps of the fission yeast membrane trafficking events, namely, at the exit from the trans-Golgi as well as at the later step of the exocytic pathway [20]. Instead, Ypt31p and Ypt32p, the homolog of Ypt3 in *S.cerevisiae*, have been reported that implicated in the exocytic pathway and mediates intra-Golgi traffic or the budding of post-Golgi vesicles from the trans-Golgi [30], [31], [32]. Thus, our results suggest that Ypt3 plays roles in transport of GPI-anchored proteins at multiple steps including the exit from the trans-Golgi as well as at the later step of the exocytic pathway. Furthermore, the localization of GFP-Gaz2 in the Δ*apm1* cells was similar to that of GFP-Ecm33, strongly suggesting that GPI-anchored proteins are transported through a clathrin-mediated post-Golgi membrane trafficking pathway that is required for the efficient transport of other secretory proteins in fission yeast. Of course, our results do not rule out the possibility that some GPI-anchored proteins might still be separately sorted from the secretory proteins in fission yeast. Further studies will be required to clarify the molecular mechanisms of membrane trafficking of GPI-anchored proteins. Given the high similarity between the fission yeast and the mammalian cells, this study may provide a basis for understanding the precise mechanism of membrane trafficking of GPI-anchored proteins in higher eukaryotes.

### Cis4 is involved in the membrane trafficking of GPI-anchored proteins

In the present study, we present several lines of evidence that suggests a role of Cis4 in membrane trafficking of GPI-anchored proteins. In our previous study, we established that zinc transporter Cis4 is implicated in Golgi membrane trafficking through the regulation of zinc homeostasis in fission yeast [11]. In this study, we first showed that the overexpression of several GPI-anchored proteins that have distinct structures, namely Ecm33, Aah3, and Gaz2, suppressed the phenotypes of the Δ*cis4* mutants. Second, there is a genetic interaction between the genes encoding Cis4 and Its8, because the *its8-1*Δ*cis4* double mutant cells were more sensitive to high temperature than that of the single mutants. Third, the Δ*cis4* mutants were sensitive to BE49385A, an inhibitor of Its8. In particular, the *its8-1*Δ*cis4* double mutants exhibited the same BE49385A-sensitive growth defects as that of the *its8-1* single mutants, while the double mutant showed defective growth similar to that of the Δ*cis4* single mutants in the zinc-deficient EMM medium. Fourth, overexpression of a majority of the multicopy suppressors of the *cis4-1* mutant complemented the MgCl_2_-sensitive phenotype of the *apm1-1* mutant. The biosynthesis of GPI anchors and its attachment to the target protein are carried out on the ER membrane, and then transported to the plasma membrane by vesicular trafficking [25]. Previous study reported that the synthesis of GPI anchors is zinc dependent both *in vitro* and *in vivo* [33], [34]. It is consistent with our results that the addition of Zn^2+^ to the medium significantly rescued the high temperature-sensitive and FK506-sensitive phenotypes of the *its8-1* mutant (Figure 5A). However, the lines of evidence as presented above suggest that the zinc transporter Cis4 may be indirectly involved in the membrane trafficking of GPI-anchored proteins, rather than involved in the synthesis of GPI anchored proteins. Consistent with this hypothesis, the subcellular localization of GFP-Ecm33 in the Δ*cis4* cells was normal and did not exhibit an abnormal accumulation in the ER in the normal medium (Figure 4D). Probably, there exists in the Golgi as yet unknown zinc-requiring components that are involved in the membrane trafficking of GPI-anchored protein, and that Cis4 delivers Zn^2+^ to the Golgi to regulate membrane trafficking of GPI-anchored proteins.

## Materials and Methods

### Strains, media, genetic and molecular biology techniques

*S. pombe* strains used in this study are listed in Table S1. The complete medium, YPD, and the minimal medium, EMM, have been described previously [35]. Standard *S. pombe* genetic and recombinant-DNA methods were performed as described previously except where noted [36]. Gene disruptions are denoted by lowercase letters representing the disrupted gene followed by two colons and the wild-type gene marker used for disruption (for example, *gaz2*::*ura4* ^+^). Gene disruptions are abbreviated by the gene preceded by Δ (for example, Δ*gaz2*). Proteins are denoted by roman letters and only the first letter is capitalized (for example, Gaz2). Tacrolimus (FK506) was obtained from Astellas Pharma (Tokyo, Japan). All other chemicals and reagents were purchased from commercial sources.

### Multicopy suppressor screen

To identify multicopy suppressors of the high MgCl_2_ sensitivity of *cis4-1* mutant, a genomic library cloned into the vector pDB248 [37] was transformed into the *cis4-1* mutant cells. The Leu^+^ transformants were replica-plated onto YPD plates containing 0.15 M MgCl_2_ and the plasmid DNA was recovered from transformants that showed a plasmid-dependent rescue. These plasmids complemented MgCl_2_ sensitivity of the *cis4-1* mutant. By DNA sequencing, the suppressing plasmids were found to belong to six classes, with one class containing the *cis4* ^+^ gene [11], and other classes containing the *ecm33* ^+^ gene, SPBC1E8.05, *aah3* ^+^ gene, *pmp1* ^+^ gene, and *trp1322* ^+^ gene (SPCC1322.03). Here we focus on the *ecm33* ^+^ gene, SPBC1E8.05, and the *aah3* ^+^ gene that encodes GPI-anchored proteins, and renamed SPBC1E8.05 gene as *gaz2* ^+^ (*gaz* for GPI-anchored protein that suppress the zinc transporter deletion).

### Knockout of the *ecm33* ^+^, *gaz2* ^+^, and *aah3* ^+^ genes

To knockout the *ecm33* ^+^ gene, a PCR-based targeted gene deletion method was prepared by the Cre-loxP-mediated marker removal procedure as described previously [38] using the sense primer 5′-CAT AGC AAG AGC AGC AAC CAA AAG AGA TCC CAA AAC TAA AGC ACC AGC AGT GAA GCC GTT AGA AGC GGC TGA GCC CAA TAG GCC GAA ATC GGC AAA ATC CC-3′, and the antisense primer 5′-GTT GTT CAA ATC ATT CGC TCT CAC TCT TCT TTT CGC CGC AGC TCG CGT ACA AGC TGC TTC CAA CTG CTC CAG CGG CCC GGT GAT GGT TCA CGT AGT GGG CC-3′. The disruption of the gene was checked using genomic Southern hybridization (our unpublished data).

To knockout the *gaz2* ^+^ gene, a one-step gene disruption by homologous recombination was performed [39]. The *gaz2*::*ura4* ^+^ disruption was constructed as follows. The open reading frame (ORF) of *gaz2* ^+^ was amplified by PCR (forward primer2232, 5′-CCG CTC GAG CAC CAT GAA GTT GTC TTT CAT TTT ATC TAC TCT CG-3′; reverse primer 2233, 5′-ATA GTT TAG CGG CCG CCA AGA AAC AAG GCA ATA GCA GAA ACA ACA CC-3′) from the genomic DNA and was subcloned into the XhoI/NotI site of BlueScriptSK (+). Then a HindIII fragment containing the *ura4* ^+^ gene was inserted into the HindIII site of the previous construct. The construct containing the disrupted *gaz2* ^+^ gene was digested with XhoI/NotI, and the *gaz2*::*ura4* ^+^ fragment was used to transform the diploids (5A/1D, Table S1). Stable integrants were subsequently cloned on plates containing the medium lacking uracil, and gene disruption by the *gaz2* ^+^ derivative containing the *ura4* ^+^ insertion was verified by genomic Southern blotting (our unpublished data).

The *aah3* ^+^ gene deletion mutant (*h^−^ leu1-32 ura4-D18 ade6-M210 aah3*::*KanMX4*) was purchased from BioNEER (South Korea) [40]. We constructed the *aah3* ^+^ gene deletion cells that are not auxotrophic for uracil or adenine by the genetic cross between wild-type cells HM123 and the above strain to make KP5075 (*h^−^ leu1 aah3*::*KanMX4*) (Table S1).

### Plasmids constructions

The *ecm33* ^+^, *gaz2* ^+^, and *aah3* ^+^ genes, respectively, together with their promoter regions were amplified by PCR using the genomic DNA of wild-type cells as a template. The primers used were summarized in Table S3. The amplified products containing the *ecm33* ^+^ or *gaz2* ^+^ genes were digested with PstI, while the amplified products containing the *aah3* ^+^ gene was digested with HindIII. All the resulting fragments were subcloned into BlueScriptSK (+), to give pKB8044, pKB7850, and pKB8147 respectively.

A series of truncated Ecm33 mutants were constructed as follows. The *ecm33* ^+^ gene lacking about 100 base pairs from the predicted region was amplified by PCR using the plasmid pKB8044 as a template, to yield fragment A, fragment B, fragment C, fragment D, fragment E, fragment F, fragment G, fragment H, fragment I, fragment J, and fragment K (Figure 3A). The primers in each mutant were derived from the upstream and the downstream regions to be deleted, as shown in Table S2.

To study the subcellular localization of Ecm33 and Gaz2, plasmids carrying GFP-Ecm33 and GFP-Gaz2 fusions, respectively, were constructed as follows. First, the sequence for GFP lacking the start codon was amplified by PCR (primers shown in Table S3) from the plasmid containing GFP carrying S65T mutation, and was subcloned into BamHI site of BlueScript SK(+). Next, BamHI site was constructed at 60 bp of Ecm33 and at 600 bp of Gaz2, respectively, by PCR technique using the primers shown in Table S3. Then, a BamHI fragment containing GFP S65T mutation was inserted into the BamHI site of the Ecm33 construct and the Gaz2 construct as described above. To obtain the chromosome-borne GFP, the fused gene GFP-Ecm33 was subcloned into the vector containing the *ura4* ^+^ marker under the control of its own promoter, and was integrated into the chromosome at the *ura4* ^+^ gene locus of KP1248 as described [17], [20]. A successful integration was confirmed by PCR and Southern blot (our unpublished data).

### Bioinformatics

Database searches were performed using the National Center for Biotechnology Information BLAST network service (www.ncbi.nlm.nih.gov) and the Sanger Center *S. pombe* database search service (www.sanger.ac.uk). Sequence alignment was performed using protein BLAST and the ClustalW program.

### Microscopy and miscellaneous methods

Methods in light microscopy, such as fluorescence microscopy that was used to observe the localization of GFP-tagged proteins and FM4-64 labeling, were performed as described [17], [20]. Krp1-RFP was expressed as described previously [41]. A fluorescent cholesterol probe, filipin was used to stain sterol as previously described [42].

### Cell extract preparation and immunoblot Analysis

Cell extract preparation was performed as described previously [43]. Protein extracts (10–20 µg/5 µl) were subjected to immunoblot analysis with anti-Ecm33 monoclonal antibody [12]. Obtained gel profiles were also visualized by CBB staining as a loading control.

## Supporting Information

Figure S1 **Mutation in the** ***chc1*** **^+^ gene causes immunosuppressant- and temperature-sensitive phenotypes.** (A) The immunosuppressant and temperature sensitivities of the *chc1-1* mutant cells. Cells transformed with the multicopy vector pDB248 or the vector containing the *chc1* ^+^ gene were streaked onto each plate containing YPD or YPD plus 0.5 µg/ml FK506, then incubated for 4 days at 27°C or 3 days at 36°C, respectively. (B) Alignment of protein sequence of *S. pombe* Chc1 with related proteins from human and *S. cerevisiae*. Sequence alignment was performed using the ClustalW program. Arrowhead points to arginine at 1615, which was mutated to a termination codon in KP555 cells by a C-to-T transition.
(TIF)

Table S1 Strains used in this study.
(DOC)

Table S2 Primers for construction of truncated Ecm33.
(DOC)

Table S3 Primers for cloning or tagging of the *ecm33* ^+^, *gaz2* ^+^ and *aah3* ^+^ genes.
(DOC)
