# Supplementary figures and images for: Studies on the Roles of Clathrin-Mediated Membrane Trafficking and Zinc Transporter Cis4 in the Transport of GPI-Anchored Proteins in Fission Yeast
Source: PLoS One. 2012 Jul 25;7(7):e41946. doi: 10.1371/journal.pone.0041946 (PMC3405024; doi:10.1371/journal.pone.0041946)

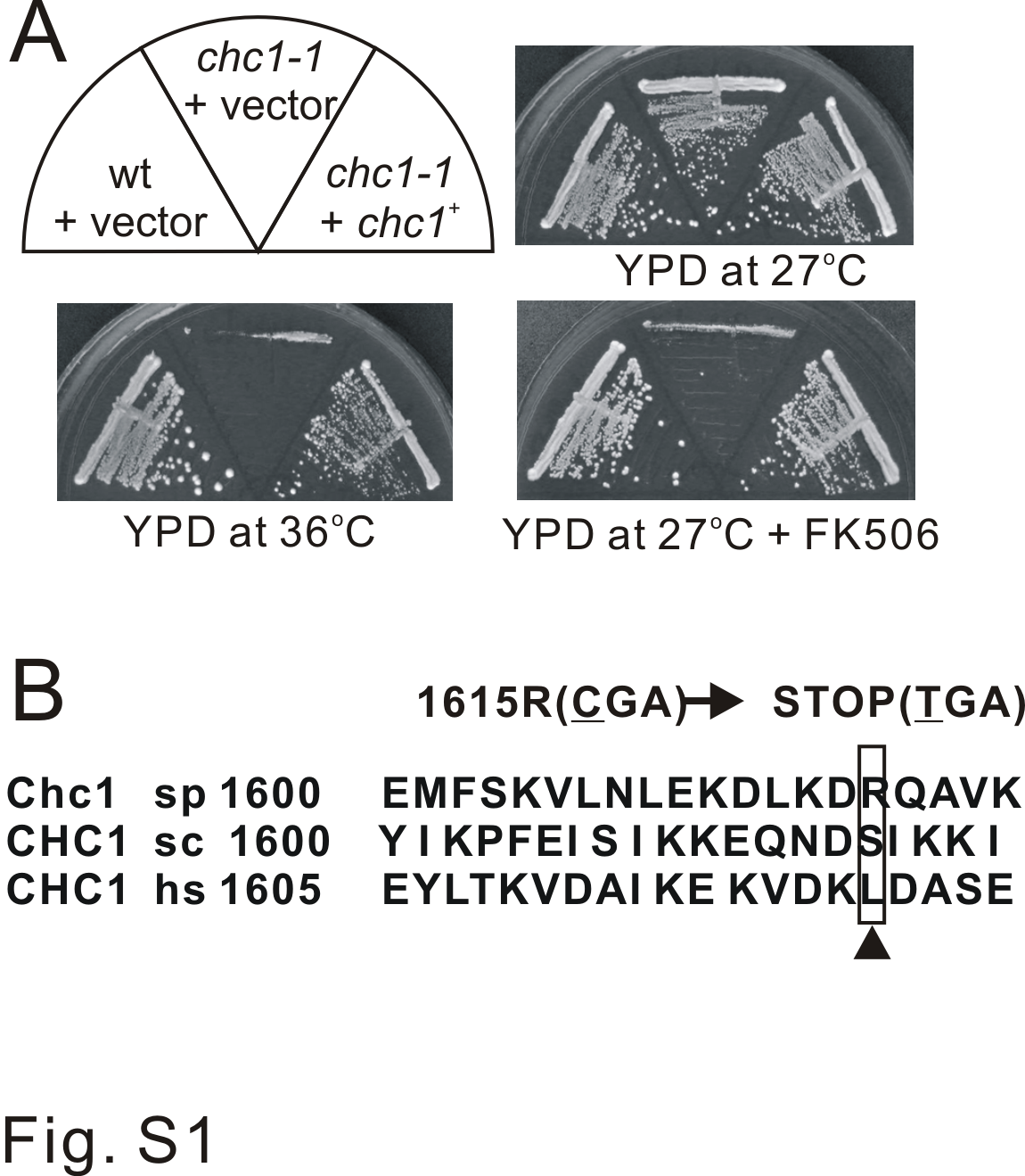

Supplement: Figure S1 — Mutation in the chc1 + gene causes immunosuppressant- and temperature-sensitive phenotypes. (A) The immunosuppressant and temperature sensitivities of the chc1-1 mutant cells. Cells transformed with the multicopy vector pDB248 or the vector containing the chc1 + gene were streaked onto each plate containing YPD or YPD plus 0.5 µg/ml FK506, then incubated for 4 days at 27°C or 3 days at 36°C, respectively. (B) Alignment of protein sequence of S. pombe Chc1 with related proteins from human and S. cerevisiae. Sequence alignment was performed using the ClustalW program. Arrowhead points to arginine at 1615, which was mutated to a termination codon in KP555 cells by a C-to-T transition. (TIF) [file pone.0041946.s001.tif]
