# Supplementary material for: Studies on the Roles of Clathrin-Mediated Membrane Trafficking and Zinc Transporter Cis4 in the Transport of GPI-Anchored Proteins in Fission Yeast
Source: PLoS One. 2012 Jul 25;7(7):e41946. doi: 10.1371/journal.pone.0041946 (PMC3405024; doi:10.1371/journal.pone.0041946)
Supplement: Table S1 — Strains used in this study. (DOC) [file pone.0041946.s002.doc]

**Supplementary information**

**Table S1. Strains used in this study.**

| Strain | Genotype | Reference |
| --- | --- | --- |
| HM123 | *h^-^ leu1-32* | Our stock |
| KP456 | *h^-^ leu1-32 ura4-D4* | Our stock |
| 5A/1D | *h^-^/h^+^ leu1-32/leu1-32 ura4-D18/ura4-D18 his2/+ ade6-M216* | Our stock |
| KP1248 | *h^-^ leu1-32 ura4-294* | Our stock |
| KP119 | *h^-^ leu1-32 ura4-D18 ppb1*::*ura4^+^* | Our stock |
| KP3051 | *h^-^ leu1-32 arg1*::*loxp* |  |
| KP457 | *h^-^ leu1-32 cis4-1* |  |
| KP680 | *h^-^ leu1-32 ura4-D18 cis4*::*ura4^+^* |  |
| KP533 | *h*^-^ *leu1 its8-1* |  |
| KP162 | *h^-^ leu1-32 ypt3-i5* |  |
| KP555 | *h^-^ leu1-32 chc1-1* | This study |
| KP356 | *h^-^ leu1-32 cis1-1/apm1-1* |  |
| KP630 | *h^-^ leu1-32 ura4-D18 apm1*::*ura4^+^* |  |
| KP4274 | *h^-^ leu1-32 arg1*::*loxp ecm33*::*arg1^+^* | This study |
| KP4588 | *h^-^ leu1-32 ura4-D18 gaz2*::*ura4^+^* | This study |
| KP5075 | *h^-^ leu1 aah3*::*KanMX4* | This study |
| KP5504 | *h^-^ leu1-32 ura4-D18 its8-1 cis4*::*ura4^+^* | This study |
| KP5563 | *h^-^ leu1-32 ura4-294* GFP-Ecm33::*ura4^+^* | This study |
| KP5571 | *h^-^ leu1-32 ura4-294 cis4*::*ura4^+^* GFP-Ecm33::*ura4^+^* | This study |
| KP5596 | *h^-^ leu1-32 ura4-294 its8-1* GFP-Ecm33::*ura4^+^* | This study |
| KP5573 | *h^-^ leu1-32 ura4-294 apm1*::*ura4^+^* GFP-Ecm33::*ura4^+^* | This study |
| KP5600 | *h^-^ leu1-32 ura4-294 chc1-1* GFP-Ecm33::*ura4^+^* | This study |
| KP5610 | *h^-^ leu1-32 ura4-294 ypt3-i5* GFP-Ecm33::*ura4^+^* | This study |
