# Supplementary material for: Studies on the Roles of Clathrin-Mediated Membrane Trafficking and Zinc Transporter Cis4 in the Transport of GPI-Anchored Proteins in Fission Yeast
Source: PLoS One. 2012 Jul 25;7(7):e41946. doi: 10.1371/journal.pone.0041946 (PMC3405024; doi:10.1371/journal.pone.0041946)
Supplement: Table S2 — Primers for construction of truncated Ecm33. (DOC) [file pone.0041946.s003.doc]

**Supplementary information**

**Table S2.** Primers for construction of truncated Ecm33. Fgt, fragment.

| Mutant | Deletion region, bp | Sequence |
| --- | --- | --- |
| Fgt A, forward primer | 70-171 | 5’-CGC GTA CAA GCT GCT TCC AAC TGC GGA TCC GGC ATC ACC ACC CTT ACT GTT AAT GG-3’ |
| Fgt A, reverse primer | 70-171 | 5’-CC ATT AAC AGT AAG GGT GGT GAT GCC GGA TCC GCA GTT GGA AGC AGC TTG TAC GCG-3’ |
| Fgt B, forward primer | 172-273 | 5’-CT GAT GCT GGT AAC TCT GGA TCC AAC GTT TCT GGT GCT TTT AAC G-3’ |
| Fgt B, reverse primer | 172-273 | 5’-C GTT AAA AGC ACC AGA AAC GTT GGA TCC AGA GTT ACC AGC ATC AG-3’ |
| Fgt C, forward primer | 274-375 | 5’-CC TCT CTT TCC TTC CCT TCT TTG AAA GGA TCC AAC TTA CAG GAG CTT CAA TTT AAC GCT GG-3’ |
| Fgt C, reverse primer | 274-375 | 5’-CC AGC GTT AAA TTG AAG CTC CTG TAA GTT GGA TCC TTT CAA AGA AGG GAA GGA AAG AGA GG-3’ |
| Fgt D, forward primer | 376-477 | 5’-GGA AGC CTA AAT CTT GCT GTT TTA CCA GGA TCC GTC ACT ACT TTC CAA GTA ACC-3’ |
| Fgt D, reverse primer | 376-477 | 5’-GGT TAC TTG GAA AGT AGT GAC GGA TCC TGG TAA AAC AGC AAG ATT TAG GCT TCC-3’ |
| Fgt E, forward primer | 478-579 | 5’-GAC GGT ATC AGT TTG GAT TCC GGA TCC GTA AGC GTA AAC TTC AGT AAA CTT TCT AAC G-3’ |
| Fgt E, reverse primer | 478-579 | 5’-C GTT AGA AAG TTT ACT GAA GTT TAC GCT TAC GGA TCC GGA ATC CAA ACT GAT ACC GTC-3’ |
| Fgt F, forward primer | 580-681 | 5’-C CAG ATT TCC GCT AAC TCT AAG GGT GGA TCC TAT TTC TCT AAC ACT ACT CTG G-3’ |
| Fgt F, reverse primer | 580-681 | 5’-C CAG AGT AGT GTT AGA GAA ATA GGA TCC ACC CTT AGA GTT AGC GGA AAT CTG G-3’ |
| Fgt G, forward primer | 682-783 | 5’-C TTG AAG AGT GCT GCT GGT AAC TTG GGA TCC TTC CCT AAC CTT ACC ACT GTT GGT GG-3’ |
| Fgt G, reverse primer | 682-783 | 5’-CC ACC AAC AGT GGT AAG GTT AGG GAA GGA TCC CAA GTT ACC AGC AGC ACT CTT CAA G-3’ |
| Fgt H, forward primer | 784-885 | 5’-CT CCT GAG CTT ACT TCC CTC AAC GGA TCC GTT CTT CTC GGT AAC TTC TCA AGC-3’ |
| Fgt H, reverse primer | 784-885 | 5’-GCT TGA GAA GTT ACC GAG AAG AAC GGA TCC GTT GAG GGA AGT AAG CTC AGG AG-3’ |
| Fgt I, forward primer | 886-981 | 5’-C CCC GTC ATC TCT GAA ATT GGT GGT GGT TTA GGA TCC CCT TGG TCC AAT GAT GAC AG-3’ |
| Fgt I, reverse primer | 886-981 | 5’-CT GTC ATC ATT GGA CCA AGG GGA TCC TAA ACC ACC ACC AAT TTC AGA GAT GAC GGG G-3’ |
| Fgt J. forward primer | 982-1077 | 5’-G ACT AAG GCT ACC AAT TTC ACT TGC GGA TCC TCC ACT GTC TCT GCT ACT AGT GG-3’ |
| Fgt J, reverse primer | 982-1077 | 5’-CC ACT AGT AGC AGA GAC AGT GGA GGA TCC GCA AGT GAA ATT GGT AGC CTT AGT C-3’ |
| Fgt K, forward primer | 1078-1179 | 5’-GCT ACT AGT TCT TAT GAT TTG TCT GGA TCC TCC CAT GAA AGC TCA GCC GCT TCT AAC GGC-3’ |
| Fgt K, reverse primer | 1078-1179 | 5’-GCC GTT AGA AGC GGC TGA GCT TTC ATG GGA GGA TCC AGA CAA ATC ATA AGA ACT AGT AGC-3’ |
