# Supplementary material for: Studies on the Roles of Clathrin-Mediated Membrane Trafficking and Zinc Transporter Cis4 in the Transport of GPI-Anchored Proteins in Fission Yeast
Source: PLoS One. 2012 Jul 25;7(7):e41946. doi: 10.1371/journal.pone.0041946 (PMC3405024; doi:10.1371/journal.pone.0041946)
Supplement: Table S3 — Primers for cloning or tagging of the ecm33 +, gaz2 + and aah3 + genes. (DOC) [file pone.0041946.s004.doc]

**Supplementary information**

**Table S3.** Primers for cloning or tagging of the *ecm33*^+^, *gaz2*^+^ and *aah3*^+^ genes.

| Primer name | Sequence |
| --- | --- |
| Ecm33 forward primer #2648 | 5’-AA CTG CAG GAG TCG AAC GCC TTG TTG TCA TGC C-3’ |
| Ecm33 reverse primer #2649 | 5’-AA CTG CAG TTA CAT AGC AAG AGC AGC AAC CAA AAG-3’ |
| Gaz2 forward primer #2380 | 5’-AA CTG CAG TTT ACC CGG TTC CCA GTT AG-3’ |
| Gaz2 reverse primer #2559 | 5’-AA CTG CAG TTA AAG AAA CAA GGC AAT AGC AG-3’ |
| Aah3 forward primer #2713 | 5’-CCC AAG CTT GGA TTT GAT ATT AGT TTA ACG GGA TCG G-3’ |
| Aah3 reverse primer #2714 | 5’-CCC AAG CTT GCA TCA GAA CAA GAA TAA GCT GTA GG-3’ |
| GFP forward primer #2619 | 5’-CG GGA TCC GGT AAA GGA GAA GAA CTT TTC AC-3’ |
| GFP reverse primer #563 | 5’-CG GGA TCC CTT GTA TAG TTC ATC CAT GC-3’ |
| Ecm33-60bp BamHI forward primer #3818 | 5’-C GCC GCA GCT CGC GTA CAA GCT GGA TCC AAC TGC TCC AGC GGC CCT TAC-3’ |
| Ecm33-60bp BamHI reverse primer #3819 | 5’-GTA AGG GCC GCT GGA GCA GTT GGA TCC AGC TTG TAC GCG AGC TGC GGC G-3’ |
| Gaz2-600bp BamHI forward primer #2638 | 5’-CC AGC TCT AGC TCC AAG TCC GGA TCC TCT TCC AGC TCT AGC TCC AAG TC-3’ |
| Gaz2-600bp BamHI reverse primer #2639 | 5’-GA CTT GGA GCT AGA GCT GGA AGA GGA TCC GGA CTT GGA GCT AGA GCT GG-3’ |
